# Cytotoxic Necrotizing Factor 1 Contributes to *Escherichia coli* Meningitis

**DOI:** 10.3390/toxins5112270

**Published:** 2013-11-22

**Authors:** Ming-Hsien Wang, Kwang Sik Kim

**Affiliations:** 1 Division of Pediatric Urology, Johns Hopkins University School of Medicine, 1800 Orleans Street, Bloomberg, 7308, Baltimore, MD 21287, USA; E-Mail: mwang33@jhmi.edu; 2 Division of Pediatric Infectious Diseases, Johns Hopkins University School of Medicine, 200 North Wolfe St, Room 3157, Baltimore, MD 21287, USA

**Keywords:** *E. coli* meningitis, CNF1, RhoGTPases, 37LRP, CNF1 secretion

## Abstract

*E. coli* is the most common Gram-negative bacteria causing neonatal meningitis, and *E. coli* meningitis continues to be an important cause of mortality and morbidity throughout the world. Recent reports of *E. coli* meningitis caused by antimicrobial resistant strains are a particular concern. These findings indicate that a novel strategy is needed to identify new targets for prevention and therapy of *E. coli* meningitis. Cytotoxic necrotizing factor 1 (CNF1) is a bacterial virulence factor associated principally with *E. coli* strains causing urinary tract infection and meningitis. We have shown that CNF1 contributes to *E. coli* invasion of the blood-brain barrier and penetration into the brain, the essential step in the development of *E. coli* meningitis, and identified the host receptor for CNF1, 37-kDa laminin receptor precursor (37LRP). CNF1, however, is a cytoplasmic protein and its contribution to *E. coli* invasion of the blood-brain barrier requires its secretion from the bacterial cytoplasm. No signal peptide is found in the CNF1 sequence. CNF1 secretion is, therefore, a strategy utilized by meningitis-causing *E. coli* to invade the blood-brain barrier. Elucidation of the mechanisms involved in CNF1 secretion, as shown in this report with the involvement of Fdx and YgfZ provides the novel information on potential targets for prevention and therapy of *E. coli* meningitis by virtue of targeting the secretion of CNF1.

## 1. Introduction

Neonatal Gram-negative bacillary meningitis continues to be an important cause of mortality and morbidity throughout the world. Case fatality rates have ranged between 15% and 40%, and approximately 50% of survivors sustain neurological sequelae [1,2,3,4,5]. A major contributing factor to such mortality and morbidity is our incomplete understanding of the pathogenesis of this disease [6,7,8,9,10,11]. 

*E. coli* is the most common Gram-negative bacillary organism that causes meningitis, particularly during the neonatal period. Both clinical and experimental data indicate limited efficacy with antimicrobial therapy alone for the treatment of Gram-negative bacillary meningitis [11,12,13]. Recent reports of neonatal meningitis caused by *E. coli* strains producing CTX-M-type or TEM-type extended-spectrum β-lactamases are a particular concern [14,15]. Drug-resistant clonal group ST131 is prevalent among *E. coli* strains causing extraintestinal infection and some carry CTX-M-type extended-spectrum β-lactamases [14]. Cerebrospinal fluid (CSF) is devoid of sufficient opsonic and phagocytic activity against meningitis-causing bacteria and eradication of the infecting pathogens from the CSF is entirely dependent on antibiotics [10,11]. An emergence of antibiotic resistance is, therefore, an important issue in the treatment of *E. coli* meningitis. Taken together, these findings indicate that a novel strategy is needed to enhance our knowledge on the pathogenesis and also to identify new targets for prevention and therapy of *E. coli* meningitis.

## 2. *E. coli* Penetration of the Blood-Brain Barrier

Several lines of evidence from human cases of *E. coli* meningitis and animal models of experimental hematogenous *E. coli* meningitis indicate that *E. coli* invasion into the brain follows a high level of bacteremia and cerebral capillaries are the portal of entry into the brain [16,17,18], but how meningitis-causing *E. coli* strains invade the blood-brain barrier and penetrate into the brain remains incompletely understood.

The development of both *in vitro* and *in vivo* models of the blood-brain barrier has facilitated our investigations on the mechanisms of microbial traversal of the blood-brain barrier, a key step required for the development of *E. coli* meningitis [6,7,8,9,10,11,19,20]. The blood-brain barrier protects the brain from microbes circulating in the blood, but meningitis-causing pathogens have been shown to penetrate the blood-brain barrier transcellularly, paracellularly and/or by means of infected phagocytic cells (Trojan-horse mechanism) [9]. Recent studies have demonstrated that meningitis-causing *E. coli* strains exhibit the ability to penetrate the blood-brain barrier by transcellular mechanism. This concept was shown by the demonstration that meningitis-causing *E. coli* strains invade human brain mircovascular endothelial cells (HBMEC) without affecting the HBMEC integrity, and penetrate into the brain without affecting the blood-brain barrier permeability and accompanying phagocytes [6,7,8,9,10,11,18,21].

## 3. Cytotoxic Necrotizing Factor 1 (CNF1) Contributes to *E. coli* Invasion of HBMEC and Penetration into the Brain

**Figure 1:**
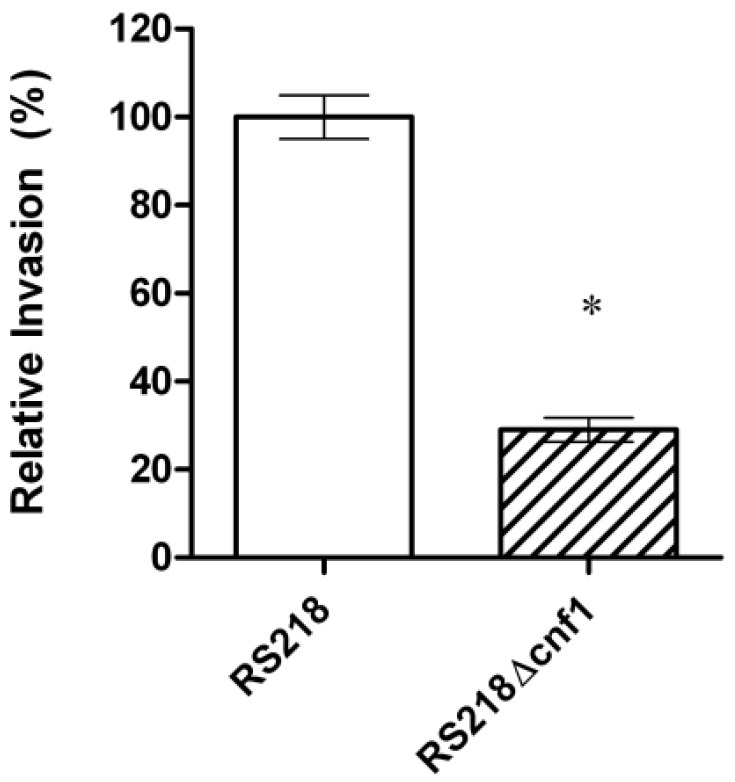
Relative invasion frequency (mean ± SD of three separate experiments in triplicate) of meningitis-causing *E. coli* strain RS218 and its CNF1 deletion mutant in human brain mircovascular endothelial cells (HBMEC) monolayer. The ΔCNF1 mutant was significantly defective (*p < 0.02 compared to the parent strain RS218, which was set at 100%) in HBMEC invasion.

CNF1 is a bacterial virulence factor associated principally with extraintestinal pathogenic *E. coli* strains causing urinary tract infection and meningitis [22]. Our previous studies using signature-tagged mutagenesis identified CNF1 that contributes to *E. coli* invasion of HBMEC monolayer and penetration into the brain in the infant rat model of experimental hematogenous *E. coli* meningitis, which mimicks the pathogenesis of *E. coli* meningitis in humans, e.g., hematogenous invasion of the meninges [23,24,25]. An isogenic mutant deleted of cnf1 was significantly less invasive in HBMEC (Figure 1) and less able to penetrate into the brain *in vivo*, and its invasive defect was restored to the level of the parent strain by complementation with respective wild-type gene [24].

CNF1 has been shown to activate RhoGTPases such as RhoA, Rac1 and Cdc42 and induce uptake of latex beads, bacteria and apoptotic bodies into non-professional phagocytes such as epithelial and endothelial cells [26,27,28,29]. The above-mentioned *in vitro* and *in vivo* effects of CNF1 in *E. coli* penetration of the blood-brain barrier, however, are dependent upon CNF activation of RhoA [24]. These conclusions were based on our demonstration that (a) the CNF1 deletion mutant exhibited significantly decreased invasion and RhoA activation compared to the parent strain in HBMEC and (b) the CNF1 mutant’s invasion frequency was restored to the level of the parent strain in HBMEC expressing constitutively active RhoA. Rac1 activation has been shown to contribute *E. coli* invasion of HBMEC, but Rac1 activation occurred in response to non-CNF1 *E. coli* factors (IbeA and OmpA) and Rac1-mediated invasion of HBMEC was shown to be under the control of STAT3 [30].

CNF1-producing *E. coli* infections have also been hypothesized to contribute to cancer development by counteracting apoptosis, and inducing pro-inflammatory cytokines’ release, COX2 expression, NF-kB activation [31]. Of interest, the host receptor for CNF1, 37-kDa laminin receptor precursor (37LRP)/67-kDa laminin receptor (67LR) is also shown to be associated with the progression of a wide variety of cancers [32,33]. It remains speculative to suggest whether CNF1-producing *E. coli* infection and/or its interaction with 37LRP/67LR may be a risk factor for cancer development such as colon cancer.

## 4. Identification of the Host Receptor for CNF1

CNF1 is an AB-type toxin, composed of the N-terminal cell binding domain and the C-terminal catalytic domain possessing a deaminase activity through the site-specific deamination of a Gln residue to Glu [34,35]. 

CNF1 has been suggested to be internalized via receptor-mediated endocytosis upon binding to a cell surface receptor [22], but the identity of its receptor was unknown. We have identified the HBMEC receptor for CNF1 by yeast two-hybrid screening of the HBMEC cDNA library using the N-terminal cell-binding domain of CNF1 as bait [33]. This receptor, 37-kDa laminin receptor precursor (37LRP) interacted with the N-terminal CNF1 and full-length CNF1 but not with the C-terminal CNF1. CNF1-mediated RhoA activation and bacterial uptake were inhibited by 37LRP antisense oligodeoxynucleotides, whereas they were increased in 37LRP-overexpressing cells, demonstrating a direct correlation between effects of CNF1 and levels of 37LRP expression in HBMEC [33]. These findings indicate that CNF1 interaction with its receptor, 37LRP, is the initial step required for CNF1-mediated RhoA activation and bacterial uptake in eukaryotic cells. The 37LRP is a ribosome-associated cytoplasmic protein and shown to be a precursor of 67-kDa laminin receptor (67LR). It is unclear how 67LR is matured and synthesized from the 37LRP, but mature 67LR is shown to be present on the cell surface and functions as a membrane receptor for the adhesive basement membrane protein laminin [36].

We have shown that incubation of HBMEC with CNF1-expressing *E. coli* up-regulates 67LR expression and recruits 67LR to the site of invading *E. coli* in a CNF1-dependent manner [37], supporting that 67LR participates in CNF1-expressing *E. coli* invasion of HBMEC. Of interest, 37LRP is also shown to be a cellular target for other CNS-infecting microorganisms, including *S. pneumoniae, N.* *meningitidis, H. influenzae* type b, dengue virus, adeno-associated virus, Venezuelan equine encephalitis virus, and prion protein [10]. The mechanisms by which the same receptor is involved in CNS penetration by different organisms, however, remain to be established.

## 5. Secretion of CNF1 across the Bacterial Inner and Outer Membrane

As indicated above, CNF1 is a key microbial factor contributing to *E. coli* invasion of HBMEC monolayer and penetration into the brain via the interaction with its receptor (37LRP) on HBMEC [24,33]. CNF1, however, is a cytoplasmic protein and execution of its contribution to *E. coli* invasion of the blood-brain barrier requires its secretion from the bacterial cytoplasm across the bacterial inner and outer membranes. No signal peptide is present in the CNF1 sequence. CNF1 secretion is, therefore, a strategy utilized by meningitis-causing *E. coli* to invade the blood-brain barrier. CNF1 is closely associated with α-hemolysin, but type 1 secretion system responsible for secretion of α-hemolysin did not affect CNF1 secretion. CNF1 secretion across the bacterial inner membrane did not involve the Sec and Tat secretion systems, and none of currently known secretion systems are involved in CNF1 secretion across the bacterial outer membrane. Recent studies have shown that CNF1 is transported to the culture supernatant in a complex with outer membrane vesicles in uropathogenic *E. coli* strains [38,39], but the underlying mechanisms remain unclear. 

## 6. Identification of Tn5 Mutants Defective in CNF1 Secretion

**Figure 2:**
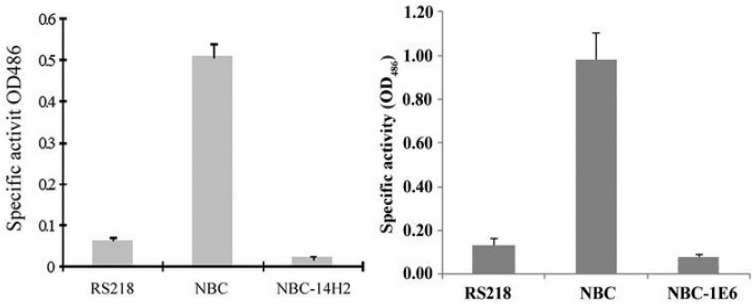
Bla activity in the culture supernatants of strain RS218 (negative control), strain NBC (positive control) and two Tn5 mutants (NBC-14H2 and NBC-1E6). The Bla activity (mean ± SD) represents the absorbance measurements of three experiments in triplicate. (modified from [40,41]).

We determined the genes involved in CNF1 secretion in meningitis-causing *E. coli*. For this study, we constructed NBC (CNF1-Bla-CAT) strain, where β-lactamase (Bla) was used as the reporter gene and translationally fused to the C-terminal of cnf1 gene in the chromosome of meningitis-causing *E. coli* strain RS218, as described previously [40,41]. In strain NBC, Bla secretion is entirely dependent upon CNF1 secretion and the secretion of CNF1 can be monitored by measuring β-lactamase activity. Our screen of Tn5 transposon mutants of NBC strain identified two mutants with defects in Bla activity but without affecting CNF1 expression (NBC-14H2 and NBC-1E6) (Figure 2). 

NBC-14H2 and NBC-1E6 were found to have Tn5 insertions in fdx and ygfZ, respectively. We have subsequently shown that ferredoxin (Fdx) is involved in CNF1 secretion across the bacterial inner membrane and YgfZ is involved in CNF1 secretion into outer membrane vesicles [40,41]. 

**Figure 3:**
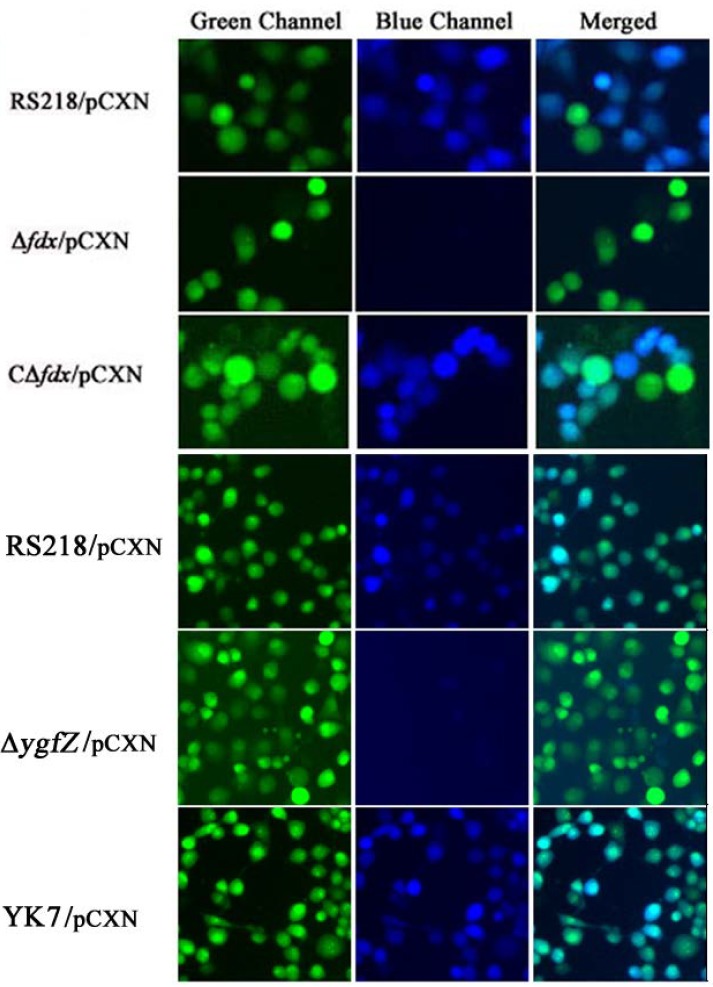
Analysis of CNF1 secretion into HBMEC monolayer. Translocation of CNF1-Bla into HBMEC induces catalytic cleavage of the CCF4 β-lactam ring, which produces an easily detectable color change in CCF4 fluorescence from green to blue emission. In the parent strain RS218/pCXN, CNF1-Bla was, as expected, successfully translocated into HBMEC, as shown by the emission of blue fluorescence. In contrast, Δfdx and ΔygfZ mutants harboring pCXN showed no blue emission, indicating no translocation of CNF1 into HBMEC. Of note, CΔfdx and YK7 represent the complemented strains of Δfdx and ΔygfZ mutants with wild type fdx and ygfZ, respectively, which showed predominant blue emission, indicating the translocation of CNF1 into HBMEC (modified from [40,41]).

We also showed that CNF1 translocation into HBMEC monolayer did not occur in the fdx and ygfZ deletion mutants, and these defects were restored by complementation with fdx and ygfZ, respectively (Figure 3). Briefly, CNF1 translocation into HBMEC monolayer was examined using *E. coli* strains harboring the plasmid pCXN, as previously described [40,41]. In pCXN, CNF1 is translationally fused with Bla, and the expression of the CNF1-Bla fusion from pCXN was induced with 1 mM IPTG. On the day of infection, HBMEC were pre-loaded with CCF4/AM dye and incubated with IPTG-treated bacteria. Non-fluorescent esterified CCF4/AM, upon entry into HBMEC monolayer is converted to fluorescent green CCF4 by cellular esterases. Translocation of CNF1-Bla into HBMEC monolayer induces catalytic cleavage of the CCF4 β-lactam ring, which produces an easily detectable color change in CCF4 fluorescence from green to blue emission. After 45 min of infection, the translocation of CNF1-Bla into HBMEC monolayer was examined under fluorescent microscope (Figure 3).

**Figure 4:**
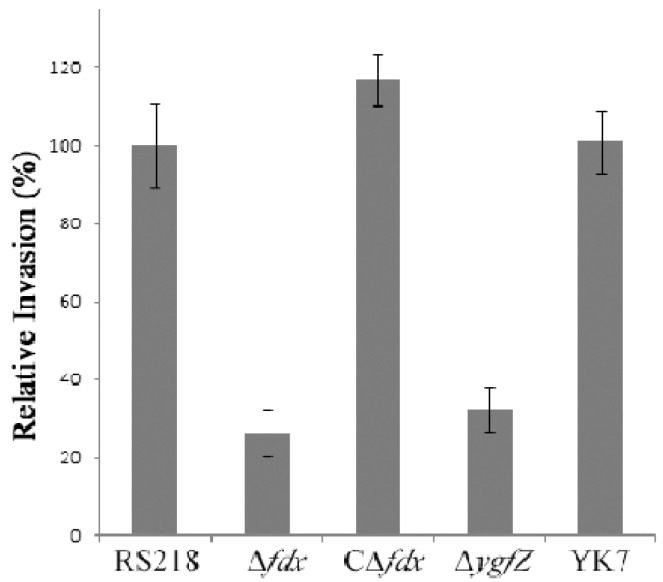
Relative invasion frequency (mean ± SD of three separate experiments in triplicate) of the deletion mutants (Δfdx and ΔygfZ) and complemented strains (CΔfdx and YK7) compared to the parent strain RS218 in HBMEC monolayer (modified from [40,41]).

We next showed that the fdx and ygfZ mutants were significantly defective in invasion of HBMEC compared to the parent strain by virtue of their failure to secrete CNF1, and their invasion defects were restored to the levels of the parent strain by complementation with respective wild type genes (Figure 4). 

Elucidation of the mechanisms involved in CNF1 secretion will, therefore, help in enhancing our knowledge on the pathogenesis of *E. coli* meningitis and also in developing a novel strategy targeting CNF1 secretion in prevention of *E. coli* meningitis. Taken together, these findings suggest that modulation of bacterial secretion systems (CNF1 secretion) is likely to represent a novel approach for investigating the pathogenesis and prevention of *E. coli* meningitis.

## 7. Modulation of Host Receptor and Signaling Molecules for Prevention of *E. coli* Invasion of the Blood-Brain Barrier

As indicated above, meningitis-causing *E. coli* penetration into the brain requires *E. coli* invasion of HBMEC, involving specific microbial-host interactions (so-called ligand-receptor interactions) and specific host cell signaling molecules [9,10,11]. We have shown that CNF1-mediated *E. coli* uptake and RhoA activation in HBMEC were inhibited by 37LRP antisense oligodeoxynucleotides, whereas they were increased in 37LRP-overexpressing cells [33]. These findings support the concept that the expression level of host cell receptor(s) dictates the fate of *E. coli* invasion of HBMEC in a bacterial ligand-dependent manner. In addition, pharmacological inhibition of the host cell receptor (e.g., 37LRP) and host cell signaling molecules (e.g., RhoA) involved in *E. coli* invasion of HBMEC was efficient in preventing *E. coli* penetration of the blood-brain barrier [42]. These findings demonstrate that pharmacological inhibition of the HBMEC receptors that interact with *E. coli* factors and host cell signaling molecules contributing to *E. coli* invasion of HBMEC might be a novel strategy for prevention of *E. coli* meningitis.

## 8. Conclusion

*E. coli* meningitis continues to be an important cause of mortality and morbidity, and recent reports of *E. coli* meningitis caused by antimicrobial resistant strains are a particular concern. These findings indicate that a novel strategy is needed to identify new targets for prevention and therapy of *E. coli* meningitis. CNF1 is a key microbial factor contributing to *E. coli* penetration of the blood-brain barrier, the essential step in the development of *E. coli* meningitis, but CNF1 is a cytoplasmic protein, and CNF1 secretion is needed to interact with the host receptor on the blood-brain barrier. CNF secretion and CNF1 interaction with the host receptor are likely to represent a novel target for prevention and therapy of *E. coli* meningitis.

## References

[1] Gladstone I.M., Ehrenkranz R.A., Edberg S.C., Baltimore R.S. (1990). A ten-year review of neonatal sepsis and comparison with the previous fifty-year experience. Pediatr. Infect. Dis. J..

[2] Unhanand M., Musatafa M.M., McCracken G.H., Nelson J.D. (1993). Gram-negative enteric bacillary meningitis: A twenty-one year experience. J. Pediatr..

[3] Dawson K.G., Emerson J.C., Burns J.L. (1999). Fifteen years of experience with bacterial meningitis. Pediatr. Infect. Dis. J..

[4] Klinger G., Chin C.-N., Beyene J., Perlman M. (2000). Predicting the outcome of neonatal bacterial meningitis. Pediatrics.

[5] Stevens J.P., Eames M., Kent A., Halket S., Holt D., Harvey D. (2003). Long term outcome of neonatal meningitis. Arch. Dis. Child. Fetal Neonatal Ed..

[6] Kim K.S. (2001). *E. coli* translocation at the blood-brain barrier. Infect. Immun..

[7] Kim K.S. (2002). Strategy of *E. coli* for crossing the blood-brain barrier. J. Infect. Dis..

[8] Kim K.S. (2003). Neurological diseases: Pathogenesis of bacterial meningitis: From bacteremia to neuronal injury. Nat. Rev. Neurosci..

[9] Kim K.S. (2008). Mechanisms of microbial traversal of the blood-brain barrier. Nat. Rev. Microbiol..

[10] Kim K.S. (2010). Acute bacterial meningitis in infants and children. Lancet Infect. Dis..

[11] Kim K.S. (2012). Current concepts on the pathogenesis of *E. coli* meningitis: Implications for prevention and therapy. Curr. Opin. Infect. Dis..

[12] McCracken G.H., Threlkeld N., Mize S., Baker C.J., Kapal S.L., Fraingezicht I., Feldman W.F., Schad U., The Neonatal Meningitis Cooperative Study Group (1984). Moxalactam therapy for neonatal meningitis due to gram-negative sepsis enteric bacilli. JAMA.

[13] Kim K.S. (1985). Comparison of cefotaxime, imipenem-cilastatin, ampicillin-gentamicin and ampicillin-chloramphenicol in the treatment of experimental *E. coli* bacteremia and meningitis. Antimicrob. Agents Chemother..

[14] Blanco J., Mora A., Mamani R., Lopez C., Blanco M., Dabhi G., Herrea A., Blanco J.E., Alonso M.P., Garcia-Garrote F. (2011). National survey of *Escherichia coli* causing extraintestinal infections reveal the spread of drug-resistant clonal groups O25b:H4-B2-ST131, O15:H5-D-ST393 and CGA-D-ST69 with high virulence gene content in Spain. J. Antimicrob. Chemother..

[15] Moissenet D., Slauze B., Clermont O., Bingen E., Arlet G., Denamur E., Merens A., Mitanchez D., Vu-Thien H. (2011). Meningitis caused by *Escherichia coli* producing TEM-52 extended-spectrum β-lactamase within an extensive outbreak in a neonatal ward: epidemiological investigation and characterization of the strain. J. Clin. Microbiol..

[16] Dietzman D.E., Fischer G.W., Schoenknecht F.D. (1974). Neonatal *Escherichia coli* septicemia—Bacterial counts in blood. J. Pediatr..

[17] Berman P.H., Banker B.Q. (1966). Neonatal meningitis: A clinical and pathologic study of 29 cases. Pediatrics.

[18] Kim K.S., Itabashi H., Gemski P., Sadoff J., Warren R.L., Cross A.S. (1992). The K1 capsule is the critical determinant in the development of *Escherichia coli* meningitis in the rat. J. Clin. Invest..

[19] Stins M.F., Gilles F., Kim K.S. (1997). Selective expression of adhesion molecules on human brain microvascular endothelial cells. J. Neuroimmunol..

[20] Stins M.F., Badger J.L., Kim K.S. (2001). Bacterial invasion and transcytosis in transfected human brain microvascular endothelial cells. Microb. Pathog..

[21] Kim K.S., Wass C.A., Cross A.S. (1997). Blood-brain barrier permeability during the development of experimental bacterial meningitis in the rat. Exp. Neurol..

[22] Boquet P. (2001). The cytotoxic necrotizing factor 1 (CNF1) from *Escherichia coli*. Toxicon.

[23] Badger J., Wass C., Weissman S., Kim K.S. (2000). Application of signature-tagged mutagenesis for the identification of *E. coli* K1 genes that contribute to invasion of the blood-brain barrier. Infect. Immun..

[24] Khan N.A., Wang Y., Kim K.J., Chung J.W., Wass C.A., Kim K.S. (2002). Cytotoxic necrotizing factor 1 contributes to *Escherichia coli* K1 invasion of the central nervous system. J. Biol. Chem..

[25] Khan N.A., Shin S., Chung J.W., Kim K.J., Elliot S., Wang Y., Kim K.S. (2003). Outer membrane protein A and cytotoxic necrotizing factor-1 use diverse signaling mechanisms for *Escherichia coli* K1 invasion of human brain microvascular endothelial cells. Microb. Pathog..

[26] Fiorentini C., Fabbri A., Flatau G., Donelli G., Matarrese P., Lemichez E., Falzano L., Boquet P. (1997). *Escherichia coli* cytotoxic necrotizing factor 1 (CNF1), a toxin that activates the Rho GTPase. J. Biol. Chem..

[27] Fabbri A., Falzano L., Travaglione S., Stringaro A., Malorni W., Fais S., Fiorentini C. (2002). Rho-activating *Escherichia coli* cytotoxic necrotizing factor 1: Macropinocytosis of apoptotic bodies in human epithelial cells. Int. J. Med. Microbiol..

[28] Doye A., Mettouchi A., Bossis G., Clément R., Buisson-Touati C., Flatau G., Gagnoux L., Piechaczyk M., Boquet P., Lemichez E. (2002). CNF1 exploits the ubiquitin-proteasome machinery to restrict Rho GTPase activation for bacterial host cell invasion. Cell.

[29] Visvikis O., Boyer L., Torrino S., Doye A., Lemonnier M., Lorès P., Rolando M., Flatau G., Mettouchi A., Bouvard D. (2011). *Escherichia coli* producing CNF1 toxin hijacks Tollip to trigger Rac1-dependent cell invasion. Traffic.

[30] Maruvada R., Kim K.S. (2012). IbeA and OmpA of *Escherichia coli* K1 exploit Rac1 activation for invasion of human brain microvascular endothelial cells. Infect. Immun..

[31] Fabbri A., Travaglione S., Ballan G., Loizzo S., Fiorentini C. (2013). The cytotoxic necrotizing factor 1 from *E. coli*: A janus toxin playing with cancer regulators. Toxins.

[32] Stallmach A., Orzechowski H.D., Feldmann P., Riecken E.O., Zeitz M., Herbst H. (1999). 32/67-kD laminin receptor expression in human colonic neoplasia: elevated transcript levels correlate with the degree of epithelial dysplasia. Am. J. Gastroenterol..

[33] Chung J.W., Hong S.J., Kim K.J., Goti D., Stins M.F., Shin S., Dawson V.L., Dawson T.M., Kim K.S. (2003). 37 kDa laminin receptor precursor modulates cytotoxic necrotizing factor 1-mediated RhoA activation and bacterial uptake. J. Biol. Chem..

[34] Flatau G., Lemichez E., Gauthier M., Chardin P., Paris S., Fiorentini C., Boquet P. (1997). Toxin-induced activation of the G protein p21 Rho by deamidation of glutamine. Nature.

[35] Schmidt G., Sehr P., Wilm M., Selzer J., Mann M., Aktories K. (1997). Gln 63 of Rho is deamidated by *Escherichia coli* cytotoxic necrotizing factor-I. Nature.

[36] Massia S.P., Rao S.S., Hubbell J.A. (1993). Covalently immobilized laminin peptide Tyr-Ile-Gly-Ser-Arg (YIGSR) supports cell spreading and co-localization of the 67-kilodalton laminin receptor with alpha-actinin and vinculin. J. Biol. Chem..

[37] Kim K.J., Chung J.W., Kim K.S. (2005). 67-kDa Laminin receptor promotes internalization of cytotoxic necrotizing factor 1-expressing *Escherichia coli* K1 into human brain microvascular endothelial cells. J. Biol. Chem..

[38] Davis J.M., Carvalho H.M., Rasmussen S.B., O’Brien A.D. (2006). Cytotoxic necrotizing factor type 1 delivered by outer membrane vesicles of uropathogenic *Escherichia coli* attenuates polymorphonuclear leukocyte antimicrobial activity and chemotaxis. Infect. Immun..

[39] Kouokam J.C., Wai S.N., Fällman M., Dobrindt U., Hacker J., Uhlin B.E. (2006). Active cytotoxic necrotizing factor 1 associated with outer membrane vesicles from uropathogenic *Escherichia coli*. Infect. Immun..

[40] Yu H., Kim K.S. (2010). Ferredoxin is involved in secretion of cytotoxic necrotizing factor 1 across the cytoplasmic membrane in *Escherichia coli* K1. Infect. Immun..

[41] Yu H., Kim K.S. (2012). YgfZ contributes to secretion of cytotoxic necrotizing factor 1 into outer membrane vesicles in *Escherichia coli*. Microbiology.

[42] Zhu L., Pearce D., Kim K.S. (2010). Prevention of *E. coli* K1 penetration of the blood-brain barrier by counteracting host cell receptor and signaling molecule involved in *E. coli* invasion of human brain microvascular endothelial cells. Infect. Immun..

